# Technique to reduce the minimum toe clearance of young adults during walking to simulate the risk of tripping of the elderly

**DOI:** 10.1371/journal.pone.0217336

**Published:** 2019-06-12

**Authors:** Jessica Beltran Ullauri, Yasuhiro Akiyama, Shogo Okamoto, Yoji Yamada

**Affiliations:** Dept. of Mechanical Systems Engineering, Mechano-Informatics and Systems, Nagoya University, Nagoya, Japan; Toronto Rehabilitation Institute - UHN, CANADA

## Abstract

The elderly gait encompasses several disorders, including a lower minimum toe clearance (MTC) to the ground, which is a potential cause of tripping and falling while walking. Devices that assist in the MTC could reduce such risks. However, the development of effective assistive methods and their evaluation in the elderly might jeopardize their safety. To address this, young adults could take the place of the elderly. We present Muscle Activity Restriction Taping Technique (MARTT) that was devised to simulate the healthy-elderly gait characteristics in the young adults, particularly the lower MTC, by restricting the activity of lower-limb muscles. Two different restriction approaches, one that restricts muscles at the shank and the other at the shank and thigh, simultaneously, were tested at different walking speeds. Both approaches achieved a reduction in the MTC, regardless of the walking speed. The MTC was reduced to a median value lower than 10.1 mm, which is within the range of the MTC values reported for the elderly. The reduction of the MTC significantly increased toe contact to the ground. With the restriction of the shank muscles, the toe-contact frequency was more than twice as that in normal walking, and with the restriction of both the shank and thigh muscles, more than five times. In addition, MARTT reproduced the lower step length, the lower single support phase, and the joint motion compensation characteristic of the elderly gait, in the youth.

## Introduction

The elderly population in the world has considerably increased; thus, several needs must be met to ensure a good quality of life for the elderly. In particular, reducing the risk of falling is a major priority because considerable evidence establishes that a fall is detrimental to their health [1–4].

Various studies have agreed that the major causes of falls in the elderly are tripping and slipping [1, 4–6]. In a three-year study of 130 individuals, Robinovitch et al. [1] reported that the main cause of fall, considering every daily activity, was incorrect weight shifting, which accounted for 48% of the total falls, followed by tripping with 21%, and slipping with 3%. The combination of the cause of fall and the activity performed immediately before falling, with the greatest number of falls, was tripping or stumbling while walking forward, accounting for 11% of falls.

Considering the trips while walking, Mills [5] suggested that the critical walking factor for fall prevention is the minimum toe clearance (MTC), defined as the minimum vertical distance between the lowest point of the toes of the swinging leg and the ground surface, during the swing phase of the gait cycle [7]. In healthy adults, at the MTC instant, the foot passes within 10 to 20 mm over the ground, in healthy adults, and travels at its maximum horizontal velocity, which is approximately three times the walking speed [7, 8]. Thus, failure to clear the ground, especially at the MTC instant, would most probably result in a fall [5].

The elderly exhibit a lower MTC with higher variability than young adults [5]. Karst et al. [9] reported an MTC of 12.9 mm in a group aged approximately 70, and Begg et al. [10] reported an MTC of 7.1 mm in a group aged approximately 72. Thus, the MTC for the elderly is likely within this range. This gait deviation in the elderly is compensated by other joints in the kinematic chain [5]. For instance, in a review article on the gait of the elderly, Prince et al. [11] reported higher hip and knee range of motion (ROM), higher knee flexion at the end of the swing phase, lower ankle ROM, and a lower ankle plantar flexion peak. Additionally, at the MTC time, Mills et al. [12] reported a higher hip and knee flexion, and lower ankle dorsal flexion. Moreover, the gait disorders in the elderly also include a decrease in walking speed, cadence, step length, and single support phase, which are adaptations for safer walking [11].

Evidence of impaired MTC in the elderly, and the subsequent high risk of falling, establish the need for studying the effect of MTC on their safety while walking, to device solutions for compensating this impairment. However, the involvement of the elderly in experimental studies on their falling risk, under several walking conditions, and the testing of assistive devices in barrier-free environments may expose the elderly to considerable danger, against the Declaration of Helsinki.

To avoid such danger, still involving humans in the study, and test assistive devices, young people can be utilized instead of the elderly, if the characteristics of the elderly to be studied can be simulated in the youth. Approaches, such as the “Aging Game” [13], the study of Wood [14] and the “Aging Suit” [15] have been introduced, which attempted to simulate the limited joint ROM, reduced dexterity, and sensory impairments of the elderly in the youth, providing insights on the challenges faced by the elderly. Moreover, in our previous study [16], we introduced Muscle Activity Restriction Taping Technique (MARTT), that could reduce the MTC of the youth, while keeping the natural motion patterns of the joints. However, insight on the feasibility of reducing the ankle ROM for reproducing the MTC characteristics of the elderly in the youth is unavailable till date.

The reproduction of the lower MTC observed in the elderly in the youth can be challenging because the central nervous system (CNS) controls the MTC with a higher priority over the other gait parameters, for high-risk tasks such as tripping [17]. For instance, in a high-risk trip activity, such as dual-task walking in the elderly [18] and chronic low back pain (CLBP) patients [17], changes on the stride length and stride time, but none in the variability of MTC, were reported, indicating that a higher priority is given to MTC control. However, in studies about single-task walking (lower risk to fall) in the elderly, a lower MTC mean value [10, 19, 20] and higher MTC variability [12, 21] have been reported. Similarly, in single-task walking in CLBP patients, a higher variability of the MTC in normal and visual-impaired walking was reported [22]. This suggests that in situations where the CNS does not detect a potential fall risk, the MTC can be reduced.

MARTT limits the transversal area of the leg muscle belly during contraction to reduce ankle flexion and lower the MTC. It is important to note that MARTT implies a blood flow reduction in the restricted muscles. Blood flow occlussion in moderation has no negative impact in the human body, in terms of blood coagulation and endothelial function [23], and can be advantageous in improving endurance [24] and increasing functional ability [23]. Due to this positive effect on the body, MARTT can reduce the MTC without triggering a control-prioritization of the MTC in the CNS.

Thus, we hypothesized that by means of MARTT, the MTC of youth can be reduced to the values observed in the elderly. In addition, the present study aimed at determining if the spatial temporal parameters and joint kinematics of the youth with MARTT correspond to those of the elderly. To assess the effects of MARTT in young adults, MARTT was applied to the shank and thigh, and evaluated at two different walking speeds. By reproducing the elderly gait characteristics in the youth, especially the lower MTC, MARTT can facilitate studies on the fall risk in the elderly and can be useful for testing ankle-assistive devices when the participation of the elderly is restricted.

## Methods

### Subjects

Ten male subjects with an average age of 22 years participated in this study. The average and standard deviation of their weight and height were 63.9 ± 8.9 kg and 1.72 ± 0.05 m, respectively. The dominant leg of all participants was the right.

Subjects were recruited from the student population of Nagoya University, in October 2015. As applicant screening did not reveal any gait disorders, all were selected. Each participant received detailed explanation on the study, in a verbal and written manner, and signed a consent form.

The Institutional Review Board of Nagoya University, Japan, approved the study and registered it under approval Number, 14-4.

### Apparatus

#### Gait measurement system

The gait motion was recorded at a frequency of 100 Hz using a motion capture system (MAC 3D system, Motion Analysis Corporation, U.S.). A set of reflective markers was attached to the body of the subject, as recommended by the motion module of the software for interactive musculoskeletal modeling (SIMM, Musculographics Inc., U.S.), used for calculating the joint angles.

**Fig 1:**
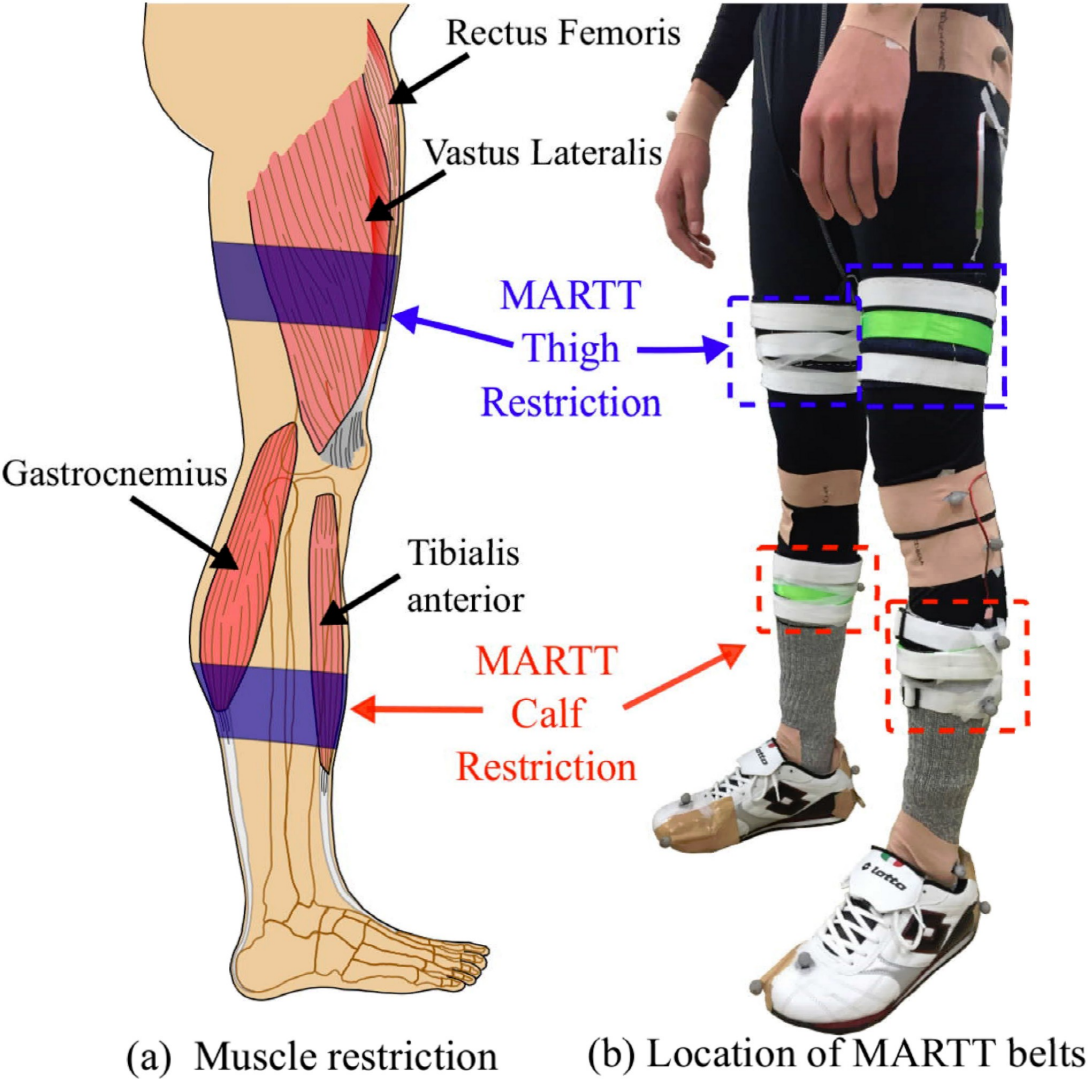
MARTT apparatus. (a) Muscles at the shank and thigh that MARTT target to restrict and (b) Position of the belts used in MARTT to restrict the transversal area of the muscle belly during contraction.

Fig 1 shows that to record the toe clearance from the ground, a marker was placed right next to each hallux, centered at the tip of the distal phalanx. To record the heel clearances from the ground, a marker was placed at the back of each calcaneus. A treadmill with a 1.4 × 0.5 m^2^ walking surface (OMEGA3, Johnson Health Tech Co., Taiwan) was used.

A non-rhythmic noise was played from an earphone during all the trials to prevent the subjects from getting distracted by the noise generated by the toe or heel contact (HC) to the ground.

In addition, four force sensors, each embedded at the shoe tip and shoe sole, respectively, of comfortable sport shoes, were installed for detecting the HC and toe off (TO) events of the gait of the subjects, without altering their natural walking patterns. Flexible force-sensing resistors (FSR-400, Interlink Electronics, U.S.) were employed for this purpose.

#### MARTT

During muscle contraction, MARTT limits the increment of the tranversal area of the muscle belly, which in turn limits the changes in the muscle length. Thereby, MARTT aims to limit the joint ROM that depends on the activity of the restricted muscles, especially at the MTC time. To decrease ankle flexion and reduce the MTC, MARTT was implemented to restrict the tibialis anterior and gastrocnemius muscles located at the shank. Additionally, to reinforce the reduction in the MTC with a lower hip and knee joint flexion, and avoid compensation in these joints, the activity of the rectus femoris and vastus lateralis muscles located at the thigh were restricted. Thus, in this study, two distinct restriction conditions were investigated.

The range of expansion of the aforementioned muscles was restricted using two pairs of MARTT belts composed of non-stretchable fabric to guarantee uniform and constant restriction, similar to our previous study [16]. Fig 1 shows that a pair of MARTT belts was placed at the end of the gastrocnemius muscle heads to restrict the shank muscles, and another pair was placed at the beginning of the rectus femoris muscle to restrict the thigh muscles. The belts were equally placed on each leg.

MARTT belts incorporate force sensors for measuring the restriction force applied to the muscles to guarantee the same force magnitude on both legs, prevent asymmetric walking, and maintain the natural gait patterns. The force was set such that it corresponds to a pressure of approximately 180 mmHg because a pressure of 160-230 mmHg [25, 26] was suggested for safely restricting the blood flow. We selected a pressure below 200 mmHg because in a study on restricted leg blood flow in older adults [23], significant muscle fatigue at such pressure was indicated.

### Protocol

Six walking trials were recorded for each subject. To avoid exhaustion, the duration of each trial was set to 6 min. Two walking speeds (regular and slow) and two muscle restriction conditions were investigated. Four trials included a combination of walking speeds and restriction conditions, whereas the other two trials included non-restricted walking at both walking speeds. A speed of 1.11 m/s (4 km/h), regarded as the natural walking speed of young adults [10], was selected as the regular walking speed, and a speed of 0.97 m/s (3.5 km/h), close to that reported in the elderly [27], was selected as the slow speed. One of the restriction conditions consisted in the application of MARTT belts at the calf only, referred to here as the C-restriction, whereas the other condition, referred to as the CT-restriction, consisted in the application of MARTT belts at both calf and thigh.

In the first two walking trials, the normal walking of the subject at each walking speed was recorded in a random order. MARTT belts then were used to implement the restriction cases (C- and CT-restriction); the implementation was randomized. For each restriction case, two walking trials corresponding to each walking speed were recorded in a random order.

The restriction forces applied to the muscles were monitored online to ensure the same magnitude in both legs, throughout the two trials, under the respective restriction cases and between cases.

### Data processing

The first minute of all gait motions in each trial, which was mostly an adaptation time to the experimental condition, was excluded from the processing. All the data were smoothed using a 6-Hz Butterworth filter. The markers-position data during each walking trial were fit to the human model, based on which the joint motions were calculated.

The HC and TO time were determined by detecting the increase and drop in the normal force on the shoe sole and shoe tip, respectively. The gait cycles were determined as the period between an HC and the next HC of the same leg, and all gait motions (clearances and joint motions) were normalized for each gait cycle. In addition, the number of toe ground contacts was manually quantified during the trials.

The toe and heel clearances from the ground were calculated as the vertical distances from the treadmill walking path to the shoe markers located immediately next to the hallux and calcaneus, respectively. From the toe clearance data, the MTC was calculated as the clearance at the instant where the toe of the swinging leg was closest to the walking surface, during the middle-swing phase. Moreover, because the MTC distribution was positively skewed and leptokurtic, as previously reported [10], the median and interquartile range (IQR) values were calculated, for each subject, as a measure of their MTC central tendency and variability.

The statistical significance of the MTC reduction in each subject was examined by the Mann-Whitney-Wilcoxon test. In addition, to determine the general tendency of the MTC (MTC between subjects), the MTC median value of each subject was normalized by the body height, and the mean, SD, median and IQR between subjects were calculated. The statistical significance of the differences of the between-subjects MTC, among conditions, was assessed using the one-tailed paired *t*-test.

To determine the significance of the gait changes because of MARTT, certain gait timing and joint motion parameters were evaluated using the one-tailed paired *t*-test, for each subject. To observe the general tendency of these parameters among the subjects, the median and IQR values between subjects were computed, and the significance of the differences was assessed by the Mann-Whitney-Wilcoxon test.

To visualize the general tendency of the toe clearance between the normal and restricted walking, the mean toe clearance pattern during the swing phase of each subject was normalized by the body height and averaged over all the subjects.

## Results

### Gait timing

The subjects’ walking symmetry was maintained during restricted walking (the gait percentage from one HC to the next of the other leg was 50.0% on an average, at 3.5 and 4 km/h during normal and restricted walking). Table 1 shows the median and IQR values for the cadence, step length and single-support phase between subjects. The step length was normalized to the subject height. According to Table 1, a lower walking speed corresponds to a lower cadence and step length. No significant differences in the cadence and step length, between normal and restricted walking, was observed. Eight subjects experienced a significantly (*p* < 0.05) lower single-support phase at C-restriction, and nine at CT-restriction, for both walking speeds.

**Table 1:** Gait timing between subjects.

Parameter	Walking Speed [km/h]	Normal walking	C-restriction	CT-restriction
Cadence [steps/min]	3.5	102.7; 95.3–107.6	101.4; 97–103.4	100.4; 96.8–105.7
	4	108; 102–109.7	104.2; 101.9–109.3	105.5; 103.3–111.1
Step Length [m]	3.5	0.57; 0.56–0.59	0.57; 0.55–0.6 (5**)	0.57; 0.55–0.59 (4**)
	4	0.6; 0.59–0.62	0.61; 0.6–0.63 (3**)	0.6; 0.59–0.64 (5**)
Single Support Phase [% stance]	3.5	64.2; 62–67.4	63.5; 61.6–64.7* (8**)	64.1; 61.8–65.2 (8**)
	4	66.4; 63.9–68.1	63.9; 62.7–67* (8**)	64.6; 63–66.1* (9**)

Median; IQR among subjects. The step length was normalized to the subject’s height. The information in parentheses indicates the number of subjects that experienced a significant reduction in the respective gait parameter.

** *p* < 0.1, and

* *p* < 0.15 indicate a significant reduction as compared to normal walking.

### Joint motion

**Fig 2:**
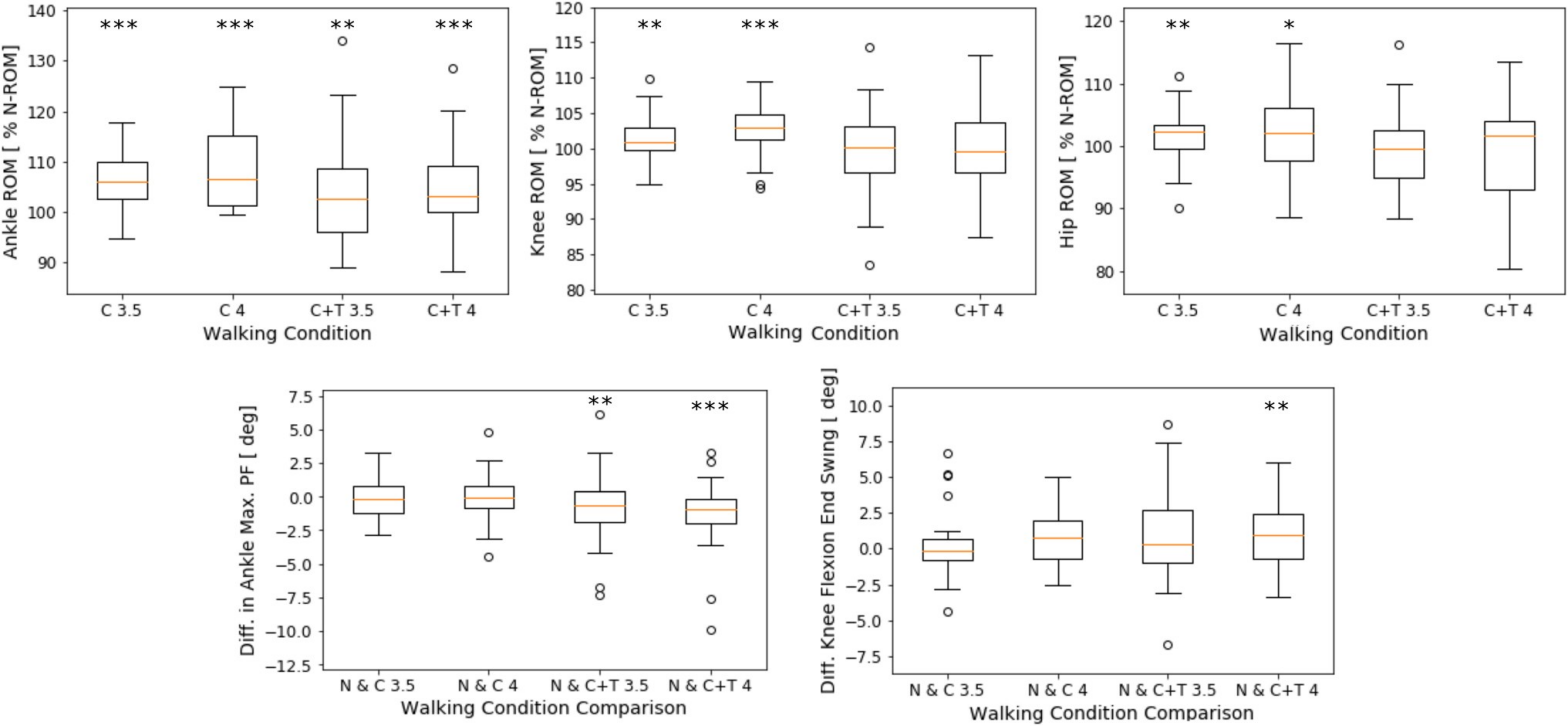
Joint motion between subjects. (Upper row) Joint ROM during C-restriction (C) and CT-restriction (C+T), at 3.5 km/h and 4 km/h walking speeds, expressed as a percentage of the ROM during normal walking. (Lower row) Difference between restricted (C, C+T) and normal walking (N) in the maximum ankle plantar flexion and knee flexion at the end of the swing phase. A negative value indicates that the respective parameter decreases during restricted walking. *** *p* < 0.01, ** *p* < 0.05, and * *p* < 0.1 indicate significant differences with normal walking. The edges of the box plot correspond to the 25-th and 75-th percentiles, and the single points (outliers) correspond to the respective parameter values that were distant from the general tendency.

In order to observe the motion changes in the ankle, knee and hip joints, the average of the joint ROM, maximum ankle plantar flexion, and knee flexion at the end of the swing phase were calculated, for each subject. The joint ROM, during C- and CT-restriction, were normalized to the respective ROM during normal walking. Fig 2 shows the between-subject distribution of the joint ROM during restricted walking, at walking speeds of 3.5 km/h and 4 km/h, expressed in percentage of the joint ROM during normal walking.

The ankle ROM increased significantly during C-restriction (*p* < 0.01), and less significantly during CT-restriction (*p* < 0.05). The knee ROM increased significantly during C-restriction (*p* < 0.05), and tended to be lower than that in normal walking during CT-restriction, but this was not significant. The hip ROM increased significantly during C-restriction (*p* < 0.1); during CT-restriction, it reduced, but was higher than that during normal walking (not significantly). The ankle maximum plantar flexion reduced significantly for both C-restriction (*p* < 0.13 at 3.5 km/h) and CT-restriction (*p* < 0.05). The knee flexion at the end of the swing phase tended to be higher for both C- and CT-restrictions at 4 km/h; it was significant during CT-restriction (*p* < 0.05).

Considering each subject independently at both walking speeds, the following number of subjects exhibited the following, at C- and CT-restriction, respectively: Three and seven subjects exhibited lower ankle ROM, six and ten subjects exhibited lower knee ROM, nine and seven subjects exhibited higher hip ROM, nine and six exhibited a lower plantar flexion peak, and ten and nine subjects exhibited higher knee flexion at the end of the swing phase.

### MTC reduction

Table 2 lists the mean, SD, median and IQR of the MTC central tendency between subjects, for each leg, during normal and restricted walking, at both walking speeds. The MTC central tendency is displayed as a percentage of the body height, and its value corresponding to the average height (1.72 m) of the subjects is listed. In agreement with the reported MTC range in young adults [7], the clearance during normal walking corresponded to a range of 10-20 mm. The MTC median value in the restricted cases was within the MTC range of the elderly reported by previous studies (e.g., 7.1 mm [10] and 12.9 mm [9]). The MTC in restricted walking was statistically proven to be lower than that in normal walking, which agrees with the relationship between the elderly and the youth [10, 12, 20].

**Table 2:** MTC central tendency (median) between subjects.

Walking speed [km/h]	Leg	Normal walking		C-restriction		CT-restriction	
		% o.b.h.	Average subject [mm]	% o.b.h.	Average subject [mm]	% o.b.h.	Average subject [mm]
3.5	Left	0.64±0.26 (0.62; 0.5–0.86)	11.1±4.4	0.40±0.31*** (0.38; 0.13–0.54)	6.9±5.4	0.47±0.41* (0.35; 0.15–0.77)	8.2±6.9
	Right	0.71±0.25 (0.68; 0.52–0.88)	12.3±4.3	0.59±0.18*** (0.59; 0.51–0.74)	10.1±3.1	0.59±0.29* (0.57; 0.35–0.76)	10.1±4.9
4	Left	0.60±0.30 (0.64; 0.34–0.86)	10.3±5.1	0.44±0.30** (0.34; 0.22–0.56)	7.5±5.2	0.41±0.30** (0.36; 0.15–0.69)	7.0±5.1
	Right	0.65±0.18 (0.63; 0.51–0.72)	11.1±3.1	0.56±0.16** (0.58; 0.49–0.68)	9.6±2.8	0.47±0.17*** (0.42; 0.33–0.65)	8.0±2.9

Mean ± SD (Median; IQR) Central tendency of the subjects’ MTC expressed in percentage of the body height (% o.b.h.), and in that corresponding to the average subject’s height, 1.72 m.

*** *p* < 0.01,

** *p* < 0.05, and

* *p* < 0.1 indicate the significant differences compared to normal walking.

**Fig 3:**
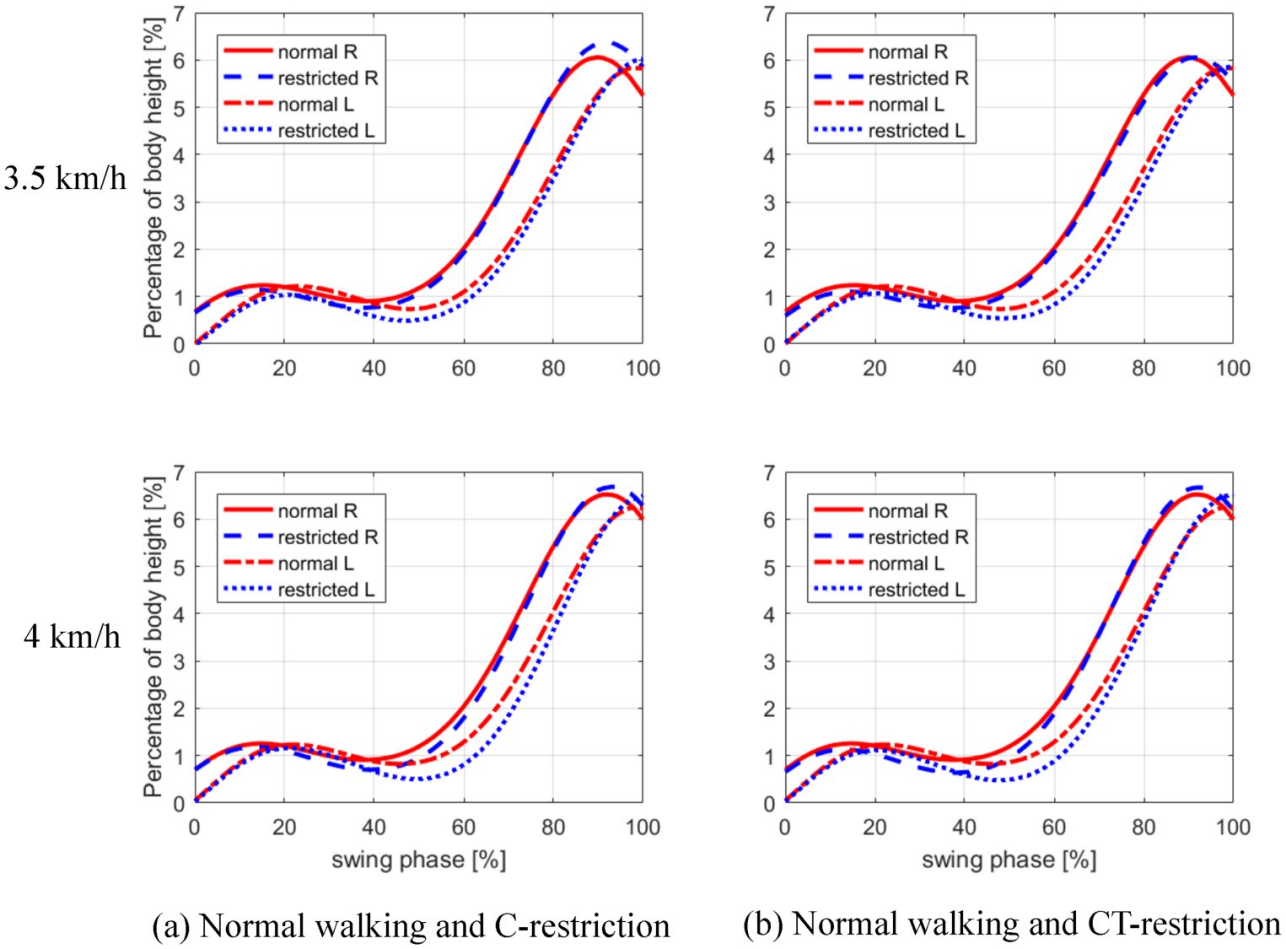
Average toe clearance during the swing phase. The MTC is the lowest point observed in the middle-swing phase, and is located between 40-50% of the swing phase. The MTC in restricted walking (C- and CT-restriction cases) is lower than that in normal walking for the right (R) and left (L) legs, at both 3.5 km/h and 4 km/h walking speeds.

The average pattern of the mean toe clearance of all the subjects is shown in Fig 3. The MTC, which is the lowest point observed in the middle-swing phase, was located between 40-50% of the swing phase, in agreement with the study of Mills et al. [5]. For each subject, the location of the MTC, during restricted walking, did not differ from that in normal walking. At the MTC instant, the swing leg and the trunk were located ahead of the support leg. The MTC, under the C- and CT-restriction cases, was lower than that during normal walking for both legs and walking speeds.

Another measure of the reduction in MTC is the number of toe contacts with the ground in the middle-swing phase. Table 3 lists the number of toe-contact events of each subject in terms of the percentage of the total gait cycles. Although toe contacts occurred even during normal walking, the occurrences under the C-restriction case were higher than those during normal walking, and even higher, under the CT-restriction case, for all subjects. The number of toe contacts with the ground was more than twice in the C-restriction case, and more than five times in the CT-restriction case, in comparison with normal walking. The ease of reducing the MTC is subject dependent, and as shown in Table 3, three subjects (B, D and E) experienced toe-contacts in almost every stride for both restriction conditions, and subject-G experienced the same frequency of toe-contacts in the strongest restriction condition, which is the CT-restriction at the walking speed of 4 km/h.

**Table 3:** Frequency of toe contacts on the ground during middle-swing phase.

Subject	Walking Speed [km/h]	Normal walking [%]		C-restriction [%]		CT-restriction [%]	
		Left	Right	Left	Right	Left	Right
A	3.5	2.06	3.44	6.60	10.76	10.53	10.18
	4	0.66	3.31	3.83	6.39	9.68	9.35
B	3.5	2.72	4.08	>90	>90	>90	>90
	4	1.15	2.29	>90	>90	>90	>90
C	3.5	0	16.78	10.84	34.97	39.66	64.14
	4	2.85	16.14	10.65	33.14	20.65	37.10
D	3.5	0	1.26	>90	>90	>90	>90
	4	0	2.24	>90	>90	>90	>90
E	3.5	1.92	9.29	>90	>90	>90	>90
	4	1.87	6.85	>90	>90	>90	>90
F	3.5	0	1.20	0.86	3.45	0.77	6.51
	4	0	0	0	0.63	18.93	11.24
G	3.5	1.04	6.92	1.69	1.69	8.06	9.52
	4	2.32	2.98	4.89	4.23	>90	>90
H	3.5	0.76	1.90	1.10	5.86	3.70	5.56
	4	0	0.66	0.95	7.94	6.51	12.70
I	3.5	0	0	0	8.94	2.27	19.42
	4	0	0.61	0.91	17.93	9.57	36.23
J	3.5	1.86	4.83	2.41	10.69	1.98	6.93
	4	0	0.66	0.65	7.14	2.68	11.74

The number of contacts is expressed as a percentage of the total gait cycles.

Additionally, we observed that the dominant leg of the subjects (right leg) tended to contact the ground less frequently than the other leg, and that the number of contacts tended to be higher at a higher speed (4 km/h).

### MTC distribution

The distribution of the MTC was not Gaussian in all the walking conditions (normal and restricted) and walking speeds. As previously reported [10], the MTC is positively skewed and leptokurtic.

**Fig 4:**
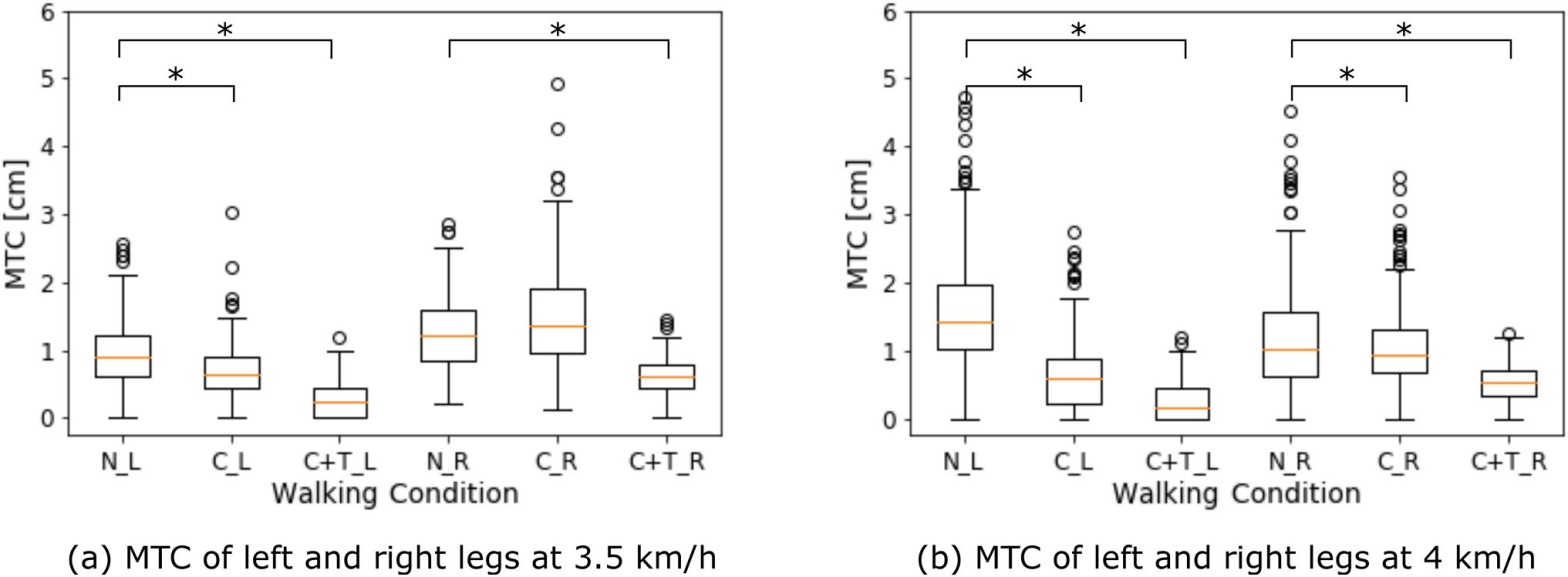
MTC median and variability (IQR) of a representative subject. The general tendency of the MTC of the subjects during restricted walking (C = C-restriction case, C + T = CT-restriction case) included a lower MTC median and IQR, compared to normal walking (N), for both right (R) and left (L) legs. * *p* < 0.01 indicates a significant lower MTC median compared to normal walking. The edges of the box plot correspond to the 25-th and 75-th percentiles, and the single points (outliers) correspond to the MTC values that were distant from the general tendency.

Fig 4 shows the main characteristics of the MTC distribution of a representative subject, because a similar tendency was observed in all the subjects. In addition to the lower MTC median, we observed that the variability (IQR) of the MTC in restricted walking tended to be lower than that in normal walking.

Table 4 shows the IQR of the MTC averaged among the subjects. The IQR corresponding to CT-restriction was lower than that in the C-restriction, and the IQR is higher at a speed of 4 km/h. The MTC IQR tends to be higher in the dominant leg, during normal and restricted walking.

**Table 4:** MTC variability (IQR) among subjects.

Walking Speed [km/h]	Leg	Normal walking [mm]	C-restriction [mm]	CT-restriction [mm]
3.5	Left	5.16 ± 1.35	4.98 ± 1.75	4.43 ± 1.33*
	Right	6.04 ± 1.31	5.01 ± 1.81*	4.75 ± 2.01*
4	Left	5.84 ± 2.49	5.37 ± 1.63	4.48 ± 1.27*
	Right	6.13 ± 1.90	4.94 ± 1.44*	4.92 ± 2.21*

* *p* < 0.1 indicate a significant reduction in the IQR in comparison with normal walking.

## Discussion

### Effect of MARTT restriction in MTC reduction

The reduction in MTC can be described by the changes in the joint angles, resulting from the restriction of the expansion range of the muscles. From the point of view of the MTC instant, the subjects exhibited the following: when the muscles at the calf alone were restricted, the MTC was reduced due to a lower dorsal flexion of the ankle (the maximum flexion decreased by 1.7° at 3.5 km/h and by 0.6° at 4 km/h, on an average). Some subjects additionally exhibited lower hip flexion, which enhanced MTC reduction. When the muscles at both the calf and thigh were restricted, the MTC was mainly reduced by the lower hip flexion (the maximum flexion decreased by 2.6° at 3.5 km/h and by 3.0° at 4 km/h, on an average). In few subjects, the restriction at the thigh also caused a reduction in the knee flexion. Thus, the principal restriction effect of the C-restriction was the reduction in the ankle flexion, and that of the CT-restriction was the reduction in the hip flexion at the MTC instant.

### Effect of the walking speed

MARTT reduced the MTC in both the C- and CT-restriction approaches, regardless of the walking speed. However, the combination of CT-restriction and a walking speed of 4 km/h (faster speed) achieved the highest reduction in MTC, as shown in Table 2.

Moreover, as it is natural for the human body to compensate restricted motions, certain subjects exhibited higher hip and knee flexion during the swing phase, when the ankle flexion was reduced by C-restriction. For these subjects, this compensation was reduced or eliminated at a walking speed of 4 km/h or when the thigh was restricted.

### Similarities and differences between MARTT-restricted youth and the elderly

MARTT can simulate a higher number of MTC values near zero clearance, as observed in the elderly, which can potentially lead to a fall. This increment in the occurrence frequency of low MTC values was reflected in a considerable number of toe contacts with the ground, for all the subjects, as listed in Table 3. Therefore, reproducing the higher ground-contact risk of the elderly is feasible using MARTT.

The difference in the gait motion between the elderly and the young adults restricted by MARTT was observed in the MTC variability. During restricted walking, the variability of the MTC of the subjects was lower than that in normal walking due to the limitation of the full range of flexion of the hip and ankle joints by applying MARTT, which prevented the exceeding of a certain maximum flexion value, in the subjects. As a result, the sporadic occurrences of high MTC values commonly observed in the elderly during walking [10] due to their loss of control of muscle contraction, were not simulated.

When both restriction cases are compared, the number of toe ground contacts was higher when both the calf and thigh muscles were restricted. Thus, the restriction at the thigh can be mainly useful for reducing the MTC of subjects, who are skillful enough to overcome the restriction at the calf muscles.

Furthermore, considering the complete gait cycle, can MARTT simulate the changes in joint motion that account for the compensation of the lower MTC, and simulate the gait characteristics caused by muscle weakness, reported in elderly walking? As mentioned in the study of Prince et al. [11], considering the complete gait cycle, the elderly exhibit the following changes in the joint motion: higher hip ROM, higher knee ROM, and higher knee flexion at the end of the swing phase, which are actions for safer walking. Additionally, lower ankle ROM, and a lower ankle plantar flexion peak were also reported, which are signs of muscle weakness in the elderly. These changes, other than a reduced ankle ROM, were also found in most subjects, when MARTT was implemented, as indicated in the Results section and Fig 2. The ankle ROM was higher after MARTT was applied because of the higher ankle dorsal flexion at terminal stance, which is due to the weakness of the gastrocnemius muscle that cannot deaccelerate the ankle dorsal flexion after mid-stance. In addition, MARTT could also reproduce the reduction of the single support phase, in the majority of subjects.

In future, we intend to assess MARTT in environmental conditions, including slopes and obstacles, to determine the effectiveness of the technique in reducing the MTC under such conditions. As suggested in the study of Khandoker et al. [8], the elderly exhibit a lower MTC median while walking on positive slope surfaces than on flat ones. Additionally, as found in the study of Hill et al. [28], falls in the elderly are likely to occur, when tripping over an obstacle. Therefore, the assessment of the relationship between the MTC and tripping in such types of challenging paths is required, and MARTT can be used as a method to further these studies.

## Conclusion

MARTT, a technique devised for reproducing the healthy-elderly gait characteristics in youth, particularly the lower Minimum Toe Clearance (MTC), was investigated in this study. This technique was implemented in the shank and thigh to limit the transversal area of muscle’ belly during muscle contraction, thereby limiting the range of motion in joints. As hypothesized, by using MARTT, the MTC median was significantly reduced to values lower than 10.1 mm, which agree with the MTC values found in the elderly, while reproducing other gait characteristics of the elderly, including lower single support phase and specific joint motion characteristics. Due to MTC reduction, the toe-contacts increased by at least double, when the shank muscles were restricted, and by five times, when both the shank and thigh muscles were restricted. The restriction of the shank muscles reduced principally the dorsal flexion of the ankle, while that of the thigh muscles reduced the hip flexion, without gait impairment.

Thus, we believe that the implementation of MARTT can broaden research on the walking of the elderly, and aid the development and evaluation of assistive devices for the MTC, by utilizing youth, in certain studies, where ethical principles restrict the participation of the elderly.

## Supporting information

S1 Table (XLSX)
